# The implicit power motive predicts decisions in line with perceived instrumentality

**DOI:** 10.1007/s11031-018-9687-1

**Published:** 2018-03-28

**Authors:** Peter Frank Stoeckart, Madelijn Strick, Erik Bijleveld, Henk Aarts

**Affiliations:** 1 Social and Organizational Psychology, Utrecht University, Heidelberglaan 1, 3584 CS Utrecht, The Netherlands; 2 Radboud University Nijmegen, Nijmegen, The Netherlands

**Keywords:** Implicit power motive, Motivation, Instrumentality, Decision-making, Intergroup context

## Abstract

Past research suggests that the implicit power motive (i.e., an unconsciously held motivational disposition to derive pleasure from having impact on others) predicts a preference to interact with individuals having submissive-looking faces. The present research extends this finding by testing whether the relation between the implicit power motive and approaching submissiveness depends on instrumentality. In two experiments, participants were assigned to a group that would ostensibly compete with another group. Within this intergroup context, they were asked to select persons as leaders or members for the in-group or the out-group. Potential leaders and members were displayed as submissive-looking or dominant-looking. Results showed that the implicit power motive predicted decisions favoring dominant-looking persons as in-group leaders, and submissive-looking persons as out-group leaders (Study 1) or in-group members (Study 2). These findings indicate that the tendency for people high in the implicit power motive to approach submissive-looking persons depends on the perceived instrumentality for gaining influence over others.

**Electronic supplementary material:**

The online version of this article (10.1007/s11031-018-9687-1) contains supplementary material, which is available to authorized users.

## Introduction

In social groups, people are often faced with the decision to choose a team member, or a team leader. For instance, members of an executive board may select a new board member, and citizens of democratic countries may elect a new president. Research suggests that such decisions may be shaped by group members’ motivational dispositions (Fodor 2010; Winter 2010). Specifically, people have been shown to have *implicit motives*, that is, motivational dispositions that operate outside of conscious awareness, and that direct behavior towards obtaining particular classes of social incentives (McClelland et al. 1989; Schultheiss 2008). The three implicit motives that are most prominent in the literature are the achievement motive, the affiliation motive, and the power motive. This research focuses on the implicit power motive (*n* Power).

People high in *n* Power “derive pleasure from having physical, mental, or emotional impact on other individuals or groups of individuals, and tend to experience the impact of others on themselves as aversive” (Schultheiss and Köllner 2008, p. 6). Accordingly, people high in *n* Power prefer job candidates who are low on assertiveness (Fodor et al. 2006). When people high in *n* Power are group leaders, they are perceived as strong leaders (Winter 2010) who act dominantly and assertively (Fodor and Riordan 1995). More than leaders low in *n* Power, leaders high in *n* Power are sensitive to compliments from their subordinates (Fodor and Farrow 1979), but they dislike conflicts among their subordinates (perhaps because this limits their own impact; Fodor 1985). In group discussions, leaders high in *n* Power tend to suppress the flow of information among group members (perhaps because this increases their own impact; Fodor and Smith 1982).

Recent studies demonstrate that other people’s submissiveness functions as an incentive for people high in *n* Power. For instance, it was shown that people high in *n* Power learned sequences faster when a face with a submissive facial expression followed the sequences than when a face with a dominant facial expression followed the sequences (Schultheiss et al. 2005). Furthermore, neuroimaging research on the role of the striatum in reward processing suggests that submissive faces serve as a reward signal for people high in *n* Power (Schultheiss and Schiepe-Tiska 2013). Moreover, recent studies by Stoeckart et al. (2017) showed that *n* Power predicts a preference for viewing submissive faces over viewing dominant faces. They developed a choice task in which participants repeatedly (and freely) decided to press one of two buttons. One button always led to the presentation of submissive faces, while the other always led to the presentation of dominant faces. As participants acquired experience with the task, and thus learned about the relationship between button presses and facial submissiveness and dominance, respectively, *n* Power became a stronger predictor of choosing the button that led to submissive faces (and hence, of not choosing the button that led to dominant faces).

The observation that *n* Power predicts a preference to interact with individuals who have submissive faces is in line with the notion that people high in *n* Power derive pleasure from having impact on others. After all, by gaining experience with social interaction in daily life, people may learn that submissive-looking people tend to be susceptible to influence. Indeed, the current assumption in implicit motive research is that people high in *n* Power approach facial submissiveness and avoid facial dominance because they associate facial submissiveness with the opportunity of taking a dominant position themselves (see also Stanton et al. 2010). In other words, facial submissiveness is perceived as *instrumental* for the acquisition of influence over others. In the current research, we examine this assumption more explicitly by testing the role of instrumentality in the preference for facial submissiveness and facial dominance, respectively.

Although submissive faces may generally signal that the incentive of influencing people is near, we suspect that this relationship is context-dependent. Submissiveness is instrumental for gaining influence when ascribed to the anticipated target of our influence (e.g., a subordinate at work, an opponent in a sports match). However, submissiveness is not instrumental for gaining influence when ascribed to the person who exerts power on our behalf (e.g., a lawyer defending our case in court, or a politician defending our national interests abroad). After all, their submissiveness would indicate that these people would likely fail to be instrumental in creating dominant position. If this reasoning is correct, then *n* Power may only predict choices favoring submissive-looking faces when these choices are instrumental for attaining influence over others. This line of reasoning is consistent with the notion that implicit motives may operate based on a *functionality principle*. This principle holds that behavior that leads to the attainment of motive-related rewards, is more likely to be learned and applied (Schultheiss and Brunstein 2002).

Several contextual factors may modify the instrumentality of specific choices and/or stimuli to attain power-related rewards. Such modifications of context could, for instance, be competitive settings where group members must select a leader of either their own group or a rival group, or must select either a leader or member of their own group (Laustsen and Petersen 2015; Spisak et al. 2012; Van Vugt and Grabo 2015). So, changes to contextual factors that affect the perceived instrumentality of submissive-looking faces may change people’s responses to those faces in a group context accordingly.

### The present research

We propose that facial submissiveness functions as an incentive to people high in *n* Power, but only when submissiveness is instrumental for attaining influence over others. We present two studies testing the purported role of instrumentality in changing how *n* Power predicts preferences in an intergroup context. For this purpose, we developed a task in which participants became members of a group and had to select individuals with submissive or dominant faces. Participants learned that these faces represented people who, if selected, would have diverging instrumentalities in providing their group with increased influence. Specifically, in Study 1, participants were led to believe that they would have to perform a competitive between-groups task. Then, they were asked to perform a decision-making task in which half of the trials pertained to the selection of an own group leader who would be tasked with influencing the rival group’s leader, thereby giving the participants’ group a competitive advantage. Additionally, the other half of the trials applied to the selection of the rival group leader. Because the own group leader would serve as an instrument through which people as a group could increase their influence in the intergroup context, we expected that *n* Power would predict more decisions favoring *dominant*-looking leaders (or fewer decisions favoring submissive-looking leaders) as own group leaders. Furthermore, we hypothesized that *n* Power would predict more decisions favoring *submissive*-looking rival group leaders. In Study 2, we asked participants to select an own group leader or an own group member. As leaders were construed as more instrumental in obtaining influence over others than members, we expected that *n* Power would predict more decisions favoring dominant-looking leaders, but not more dominant-looking members.

One could argue that this decision-making task is inappropriate to study implicit motives. After all, research typically shows that implicit motives do not predict explicit (or *declarative*) decisions, that is, decisions “that tap into a person’s verbally represented sense of self and the attitudes, judgments, decisions, and goals associated with it” (Schultheiss and Köllner 2008, see also; Biernat 1989; McClelland et al. 1989; Schultheiss 2001; Slabbinck et al. 2013; Spangler 1992). Implicit motives are thought to predict only *non-declarative* behavior, that is, behaviors “that are not accessible to, or controlled by, a person’s self-concept or verbally represented intentions” (Schultheiss and Köllner 2008). Although our decision-making task requires that participants make explicit choices, we argue that it measures non-declarative behavior, and hence, is responsive to implicit motives. Research has shown that implicit motives respond to nonverbal stimuli, particularly facial stimuli (e.g., Schultheiss 2001; Stanton et al. 2010), and that *n* Power modulates immediate affective reactions towards dominant and submissive facial expressions (Schultheiss and Schiepe-Tiska 2013). Immediate affective reactions may directly guide explicit choices, particularly in the absence of other diagnostic information about the choice alternatives, and even when people are unaware of the causes of the affective reactions (e.g., Bechara and Damasio 2005; Winkielman et al. 2005). Indeed, various models on human motivation and decision-making converge on the idea that immediate affective reactions are the starting point for explicit decisions, even deliberate ones (e.g., Fazio 1990; Gawronski and Bodenhausen 2006; Strack and Deutsch 2004; Zajonc 1980). Thus, we assume that the participants in our experiments make choices based on immediate affective reactions to the faces, without being aware of the implicit motives underlying these affective reactions. In that sense, the task measures non-declarative behavior. Previous research using a similar choice task showed that implicit motives, and not explicit motives, indeed predicted explicit choices between submissive and dominant faces (Stoeckart et al. 2017).

## Study 1

Study 1 was designed to test the hypothesis that the extent to which *n* Power predicts decisions for specific face types depends on whether these faces were construed as instrumental to the person’s ability to influence others. This was operationalized by representing these faces as belonging to potential leaders of the own versus a rival group, within a competitive between-group setting. Specifically, it was hypothesized that *n* Power would predict decisions favoring submissive faces when these faces were said to represent potential rival group leaders, but that this effect would disappear or even reverse for faces said to represent potential own group leaders. Whether the effect would disappear or reverse was hard to predict a priori. On the one hand, previous research typically found that people high in *n* Power prefer submissive faces. The own group leader context may merely counteract this default preference, which would lead to a null-effect. On the other hand, our instrumentality account predicts that the effect will fully reverse in the own group leader context, as dominant leaders are more instrumental for attaining influence than submissive leaders.

### Method

#### Participants

We did not do an a-priori power analysis because we used a novel paradigm to examine a hypothesized effect with an unknown effect size. Following recommendations from Simmons et al. (2013), we decided on a sample size of at least *N* = 50. Fifty-four students were recruited for Study 1 in exchange for a monetary compensation or partial course credit. We excluded the data from one participant who suffered a computer crash. This left 53 participants (35 female) with an average age of 20.94 years (*SD* = 2.30) for analyses. The study used a group (own group versus rival group) within-subjects design with *n* Power as continuous predictor. For all data and materials, see https://data.mendeley.com/datasets/dznks8yfhs/draft?a=e278cd15-8b6f-453e-b47f-0cf6299a150b.

#### Materials and procedure

The study was introduced as the first session of a two-part study, with the second session taking place the following week. Before starting the study, participants made an appointment for this second session by indicating a preferred date. Then, the study started with assessing *n* Power. This was followed by a group formation and leader context task, a choice-task of faces and, finally, additional measures. Although ‘groups’ and future ‘group competitions’ were mentioned, participants were run in individual sessions.

##### Measurement of *n* Power

*n* Power was measured with the Picture Story Exercise (PSE). The PSE is a reliable, valid and stable measure of implicit motives, and constitutes the most commonly used task for measuring said motives (Latham and Piccolo 2012; Pang 2010; Ramsay and Pang 2013; Pennebaker and King 1999; Schultheiss and Pang 2007; Schultheiss and Schultheiss 2014; Schultheiss et al. 2009). Importantly, *n* Power as measured by the PSE shows no correlation with explicit measures of the same construct (Köllner and Schultheiss 2014). During this task, we presented participants with six pictures of ambiguous social scenarios, one by one, for ten seconds each. After viewing each picture, participants were asked to write a complete, imaginative story about the picture—an imaginative story with a beginning, a middle, and an end. Participants were asked to try to portray who the people in each picture were, what they were feeling, thinking, and wishing for, what led to the situation depicted, and what would happen subsequently. Participants were given 2–4 min per story. The pictures portrayed two boxers; two trapeze artists; two women in a laboratory; a couple by a river; a couple in a nightclub; a ship captain and passenger. These pictures are often used in the PSE and constitute the most strongly recommended pictorial stimuli (Pang and Schultheiss 2005; Schultheiss and Pang 2007).

In accordance with Winter’s (1994) *Manual for scoring motive imagery in running text*, an experienced implicit motives rater scored the stories for power motive imagery for every occurrence of any strong and/or forceful actions with an inherent impact on other people or the world at large; attempts to control or regulate others; attempts to influence, persuade, convince, make or prove a point; provision of unsolicited help, advice or support; attempts to impress others or the world at large; (concern about) fame, prestige or reputation; or any strong emotional reactions in one person or group of people to the intentional actions of another. We also coded affiliation and achievement motive imagery, as Winter’s scoring manual prescribes the simultaneous coding of power, achievement and affiliation motivation. Furthermore, this allowed us to compare the hypothesized predictive value of power motivation with the non-hypothesized predictive value of affiliation and achievement motivation. The experienced condition-blind rater had previously obtained a confidence agreement exceeding .85 with expert scoring (Winter 1994). To determine the reliability of the ratings of the first rater, a second condition-blind rater who also obtained a confidence agreement exceeding .85 with expert scoring (Winter 1994) re-scored all PSE stories. The interrater reliability for *n* Power, as assessed by the Intraclass Correlation Coefficient (ICC, Pang 2010), was acceptable for Study 1: ICC = .69, and for Study 2: ICC = .84. The ICC for the implicit achievement and affiliation motives were acceptable as well (Study 1: .89, .95, respectively; Study 2: .85, .95, respectively). The scores of the first experienced rater were used for analyses. The absolute number of power motive images (*M* = 5.47, *SD* = 2.89) was slightly right-skewed (skewness = .55, *SE* = .33) and correlated significantly with story length in words (*M* = 561.77, *SD* = 154.73), *r*(51) = .55, *p* < .001. In accordance with recommendations (Schultheiss and Pang 2007), a regression for word count was therefore conducted, whereby power motive scores were converted to standardized residuals.

##### Group formation and leader context task

Participants were subsequently told that the rest of the study related to a competitive between-groups task. They were led to believe this task would take place in the second session—in reality, the second session only included the re-test of our implicit (PSE) and explicit (Personality Research Form; PRF) motive measures (test–retest reliability analyses are reported as Supplementary Material. The Supplementary Material file is posted online together will all data and materials, see Participants). Before being able to conduct this task, however, participants were informed that groups needed to be formed and group leaders needed to be appointed. Leaders were purported to serve the role of influencing the other group’s leaders to give their own group a competitive advantage. Consequently, the two subsequent tasks were stated to have the purpose of first dividing participants into groups and secondly, deciding who would be the leaders of these groups.

For the supposed distribution of participants into groups, we used the minimal group paradigm (Hertel et al. 2002; Mullen et al. 1992). Across seven trials, participants were presented with numerous shapes on the screen and then had to indicate how many shapes they thought had been presented. These answers were stated to indicate people’s perceptual tendencies, on which the group distribution would be based. We presented 39, 48, 57, 66, 75, 84, or 93 randomly colored shapes in a random order. Each shape’s position on the screen was randomly determined, while overlapping with other shapes was avoided. Shapes were either circular, rectangular or triangular in equal numbers. After three seconds, the shapes disappeared and participants had to estimate the number of shapes that had been present on the screen. The next trial started immediately after a response was given. After completing the task, participants were randomly distributed into either the “detailed-perceivers” (*n* = 27) or “global-perceivers” (*n* = 26) group. Detailed-perceivers were described as generally being more focused on details, whereas global-perceivers were described as generally being more focused on the bigger picture. These groups were then indicated to be the groups that would compete in the second session’s competitive task and for which group leaders had to subsequently be selected.

##### Choice of leader faces

**Fig. 1:**
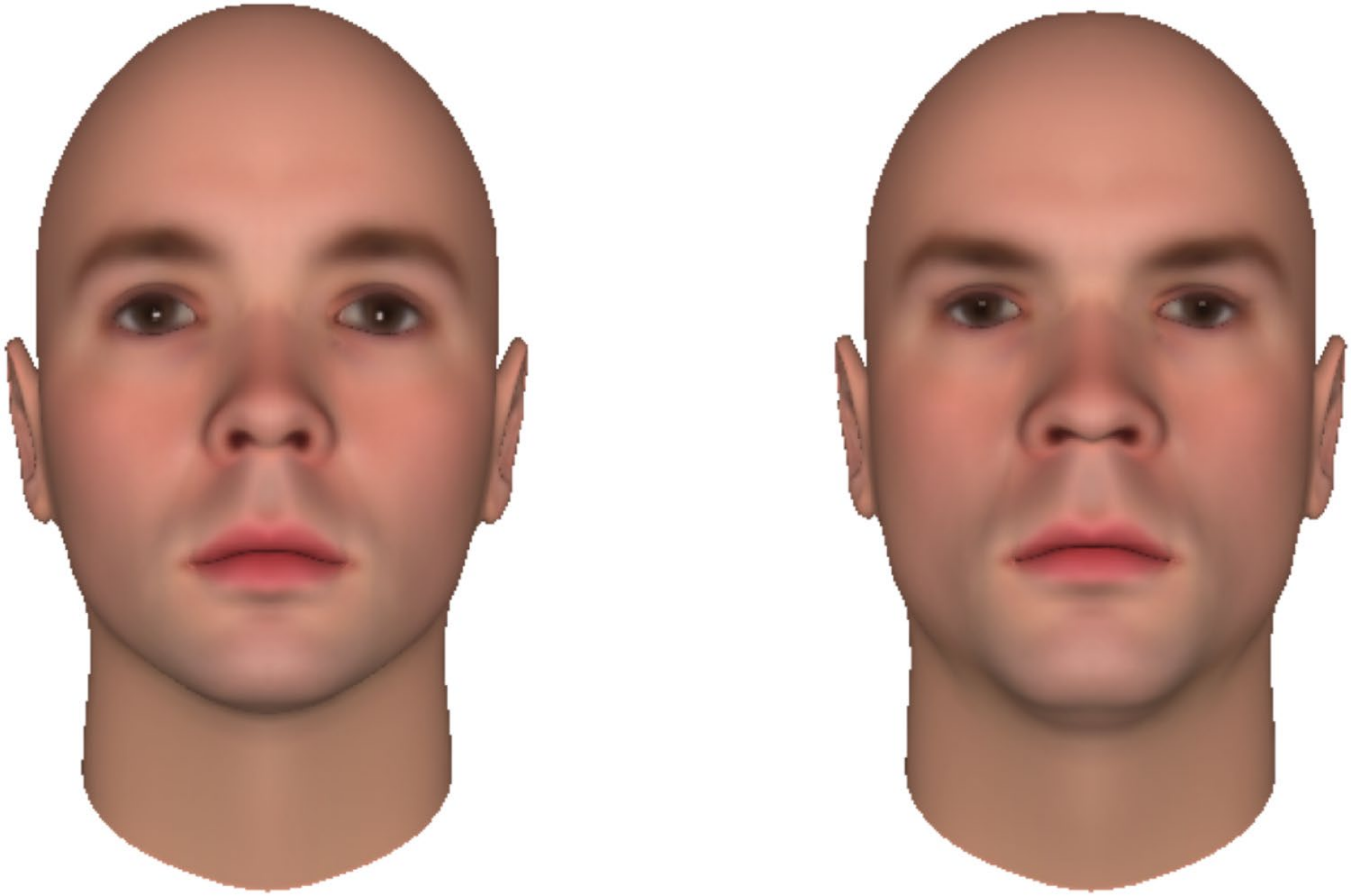
Examples of submissive (left) and dominant faces (right) used in Study 1 and Study 2. Faces were taken from the Dominance Face Data Set (Oosterhof and Todorov 2008)

Group leaders were said to be selected based on the subsequent choice task. During each trial, participants first saw an instruction for two seconds in the middle of the screen. This instruction indicated whether the succeeding leader-face choice would relate to either their own or rival group. Then, two faces were presented on the left and right of the screen respectively and participants had to indicate which of these persons they would prefer to become the leader of the respective group by pressing the A (left face) or L (right face) key on the computer keyboard. Faces were taken from the Dominance Face Data Set (Oosterhof and Todorov 2008). This is a stimulus set of 25 different Caucasian male faces with a direct gaze, computer-generated with FaceGen 3.1 software. Two versions of the 25 faces were used; one version 2SD below and one version 2SD above the mean dominance level, representing the submissive and dominant faces, respectively (see Fig. 1 for examples of the face stimuli). On each trial, submissive and dominant faces were randomly selected without replacement from the lists of 25 submissive and 25 dominant faces. Note, however, that the selection without replacement started anew after all 25 faces of the list had been selected. Hence, the same submissive and dominant faces were used in several trials. The variation in dominance is based on facial features, not facial expressions (as was the case in Schultheiss et al. 2005; Schultheiss and Schiepe-Tiska 2013). The fact that the faces were computer-generated modifications was explained to participants as following from the need to maintain potential leaders’ anonymity. Hence, the faces were said to closely represent the actual potential leaders’ faces, but not literally be their faces.

There were 80 trials in total, 40 relating to the own group and 40 relating to the rival group. The 40 trials in each group consisted of 10 submissive-left/submissive-right, 10 submissive-left/dominant-right, 10 dominant-left/submissive-right, and 10 dominant-left/dominant-right trials.^1^ The different trials were presented in random order, with randomization being limited to result in equal numbers of own or rival group trials per half of the task and each option within this group (i.e., submissive/submissive faces, submissive/dominant faces, dominant/submissive faces, dominant/dominant faces) being selected randomly without replacement. The equal face trials (i.e., submissive/submissive faces and dominant/dominant faces) were included to obscure our specific interest in the comparison between dominant and submissive faces. The same faces were used for the own group and the rival group. We did not consider this a problem given the hypothetical nature of the choice task (i.e., on each trial, participants were asked to select which of the two persons they would prefer to become the leader).

After participants had indicated their choice, the faces disappeared. This was followed by a randomly determined 200–700 ms inter-trial interval, after which the next trial started anew. Participants were informed when they completed half the task. The main dependent variable was the proportion of choices for submissive faces in the different face category trials (which is reversely related to the proportion of dominant faces). Though we were mainly interested in people’s choices (for dominant-looking versus submissive-looking leaders), we also analyzed the time participants took to make these choices in both studies. These analyses are reported in the online Supplementary Material.

##### Additional measures

The choice task was followed by a manipulation check, during which participants were first asked to indicate to which group (detailed-perceivers versus global perceivers) they had been allocated. Then, participants were asked to indicate to which extent they agreed with this allocation on a 7-point Likert scale ranging from 1 (*not at all*) to 7 (*very much*). The following eight randomly ordered questions used the same Likert scale, requesting of participants to indicate to which extend they identified [felt a connection with] the own [rival/detailed-perceivers/global-perceivers] group. The questions relating to the group participants had been allocated to showed a high reliability (α = .89) as did the questions relating to the group that participants had not been allocated to (α = .83). We therefore collapsed these two groups of questions into two separate variables.

Participants were then asked five 7-point Likert questions regarding how motivated they were to complete the tasks as well as possible, and how difficult, important, fun and annoying they considered this to be. Subsequently, they were asked 36 randomly ordered questions from the shortened PRF (Jackson 1974). This questionnaire consisted of three 12-item subscales relating to how achievement—(α = .75), power—(α = .83), and affiliation-motivated (α = .77) people considered themselves to be. Lastly, participants were asked several demographic and open questions.

#### Preparatory data analysis

No participant’s data were excluded from the analyses.

### Results

#### Manipulation check

We first assessed whether participants’ random allocation to the different groups (i.e., detailed- vs. global-perceivers) had resulted in divergent feelings of group membership. Notably, all participants accurately recalled the group they had been allocated to. Furthermore, a one-sample *t* test indicated that agreement with the group allocation (*M* = 4.81, *SD* = 1.26) differed significantly from the midpoint of the scale, *t*(52) = 4.75, *p* < .001. A repeated measures ANOVA with the questions regarding the identification and connection with the own versus rival group as dependent variables and condition (i.e., detailed- vs. global-perceivers) as independent variable indicated that participants significantly favored the own group over the rival group, *F*(1, 52) = 38.86, *p* < .001, $\eta _{{\text{p}}}^{2}$ = .43. This effect did not differ between-conditions, *F* < 1.

#### Main analyses

We hypothesized that the extent to which *n* Power predicts choices for specific face types would depend on whether these faces were construed as belonging to potential leaders of the own versus a rival group. Specifically, it was hypothesized that *n* Power would predict decisions favoring relatively submissive faces when these faces were said to represent potential rival group leaders, but that this effect would disappear or even reverse for faces said to represent potential own group leaders. In a repeated measures ANCOVA, we analyzed the proportion of decisions favoring submissive faces using group (own group versus rival group) as repeated measures and *n* Power as continuous predictor. This analysis firstly observed a significant main effect of group, *F*(1, 51) = 9.01, *p* = .004, $\eta _{{\text{p}}}^{2}$ = .15. More submissive faces were selected in the rival (*M* = 63.71%, *SE* = 2.75) than in the own group trials (*M* = 49.11%, *SE* = 3.10). One-sample t-tests indicated that the proportion of decisions favoring submissive faces differed from chance level (50%) in the rival group, *t*(52) = 4.76, *p* < .001, but not in the own group *t* < 1. This indicates that people generally prefer leaders of rival groups to appear submissive. The main effect of *n* Power was not significant, *F* < 1.

**Fig. 2:**
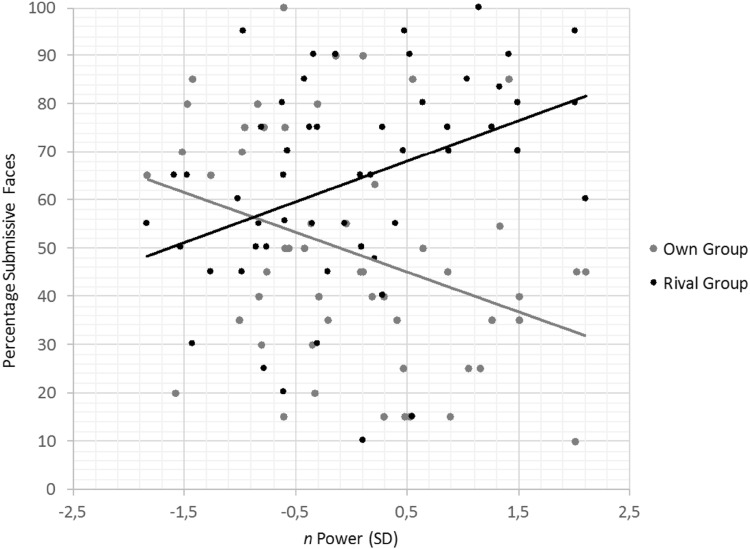
Percentage of choices leading to submissive (vs. dominant) faces as a function of *n* Power and group (own vs. rival) in Study 1

More importantly, the hypothesized interaction between group and *n* Power was significant, *F*(1, 51) = 11.36, *p* = .001, $\eta _{{\text{p}}}^{2}$ = .18. As can be observed in Fig. 2, *n* Power positively predicted decisions favoring submissive faces for the rival group, *r*(51) = .39, *p* = .004, but negatively for the own group, *r*(51) = − .35, *p* = .012.

#### Other motives

In accordance with the general literature (Köllner and Schultheiss 2014), no significant correlation was observed between *n* Power the implicit and the explicit power motive, *r*(51) = − .039, *p* = .783. To investigate whether the aforementioned predictive relation between *n* Power and decisions as a function of group was specific to *n* Power, we replaced this motive for the explicit power motive as continuous predictor in the ANCOVA with group (i.e., own vs. rival) as repeated measures. This analysis revealed no main or interaction effect of the explicit power motive, *F*s < 1. The same held true when we instead entered either the implicit achievement or affiliation motive. Hence, the predictive relation appears to have been specific to *n* Power.

### Discussion

In line with our hypothesis, results showed that *n* Power divergently predicted leader choices based on the leader candidate’s instrumentality to acquire influence. These results support the idea that implicit motives, *n* Power in particular, are not bound to specific styles of behavior or preferences for specific stimuli. Instead, they operate in a way in which the perceived instrumentality of the behavior is taken into account.

Although these results confirmed our prediction that implicit motives operate based on perceived instrumentality, several alternative explanations remain. First, it is also possible that this predictive relationship occurred because people relatively high in *n* Power are more prone to ascribe the concept of dominance to themselves, and are therefore more likely to choose a dominant-looking person to be included in their own group. For Study 2, then, we chose to keep group membership constant in the design. That is, both roles for which participants had to select a person (previously own versus rival group leader) were own group members. However, half of the decisions were stated to relate to the group leader, whereas the other half was stated to relate to a regular group member. As only the leader was stated to be instrumental in obtaining influence over others, *n* Power should still divergently predict decisions favoring dominant versus submissive faces as a function of condition. In other words, whereas Study 1 investigated whether the predictive relationship between *n* Power and decisions would depend on the orientation of the power (i.e., favoring versus disfavoring own group), Study 2 investigates whether the predictive relationship between *n* Power and decisions would depend on the person’s function in the group (i.e., influential versus not).

## Study 2

### Method

#### Participants

We determined the planned sample size based on findings from Study 1. Specifically, Study 1 revealed *r* = .39 for the association between *n* Power and choices for the leader of the rival group. Assuming this effect size, *N* = 46 would be needed to detect the effect with alpha = .05, and power at .80. Thus, we again decided on a sample size of at least *N* = 50. Fifty students (36 female) with an average age of 21.94 years (*SD* = 2.88) participated in the study in exchange for a monetary compensation or partial course credit. The study used a within-subjects design with *n* Power as continuous predictor.

#### Materials and procedure

Study 2 almost fully mimicked the procedure of Study 1, with a few changes. The first deviation occurred after the PSE,^2^ when it was mentioned that participants would first be divided into groups and would then have to decide who would become their own group leader and who would become an own group member. It was specifically stated, and reiterated in the specific task instructions, that the selection of the leader and member were independent. Hence, that the selection of one person as group leader would not automatically mean that another person would become a regular group member, which would confound the two conditions. Instead, it was stated that the current group already consisted of nine people, including the participant him/herself. Based on the decisions for group leader, one person would be added to the group as group leader. Based on the decisions for group member, one person would be added to the group as group member. All potential candidates were stated to be of the congruent minimal group (i.e., detailed- or global-perceivers).

For the choice task itself, the procedure remained the same, except for the condition wherein it was previously stated that the decisions would determine who would become the leader of the rival group now stating that the decisions would determine who would become an additional member of the own group.

### Results

#### Manipulation check

In accordance with Study 1, all participants had accurately recalled the group they had been allocated to. Furthermore, a one-sample *t* test again indicated that agreement with the group allocation (*M* = 4.98, *SD* = 1.02) differed significantly from the midpoint of the scale, *t*(49) = 6.79, *p* < .001. A repeated-measures ANOVA with the questions regarding the identification and connection with the own versus rival group as dependent variables and condition (i.e., detailed vs. global perceivers) as independent variable indicated that participants again significantly favored the own group over the rival group, *F*(1, 48) = 14.40, *p* < .001, $\eta _{{\text{p}}}^{2}$ = .23. This effect did not differ between-groups, *F* < 1.

#### Main analyses

We hypothesized that *n* Power should predict relatively more decisions favoring the submissive faces in the member condition compared to the leader condition. In a repeated measures ANCOVA, we analyzed the proportion of decisions favoring submissive faces using role (leader versus member) as repeated measures and *n* Power as continuous predictor. This analysis first revealed a significant main effect of role, *F*(1, 48) = 88.23, *p* < .001, $\eta _{{\text{p}}}^{2}$ = .65. One-sample *t* tests comparing the decisions per role with chance level (50%) indicated that decisions for the leader (*M* = 37.40%, *SD* = 20.41) were significantly less likely to favor the submissive face, *t*(49) = − 4.37, *p* < .001, with the opposite being true for the member (*M* = 71.60%, *SD* = 16.89), *t*(49) = 9.05, *p* < .001. Note that participants in Study 2 thus preferred dominant own leaders, an effect which had not been significant in Study 1.

**Fig. 3:**
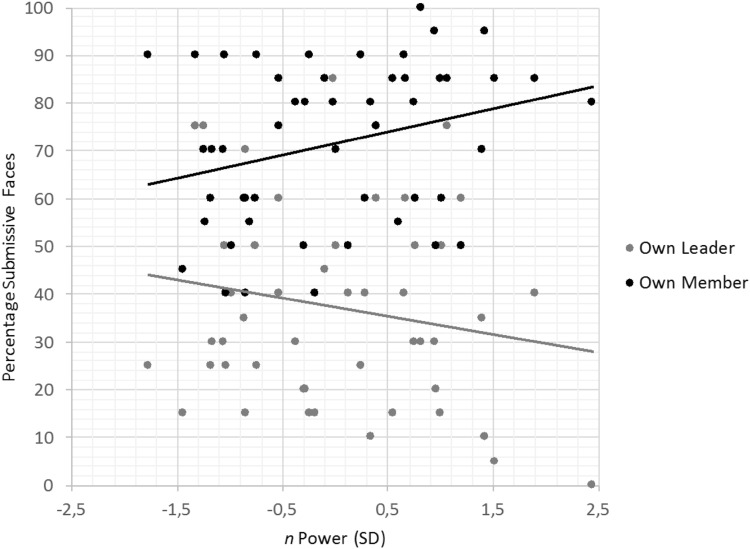
Percentage of choices leading to submissive (vs. dominant) faces as a function of implicit power motive and role (leader versus member) in Study 2

In accordance with the hypothesis, a significant interaction was observed between role and *n* Power, *F*(1, 48) = 5.43, *p* = .024, $\eta _{{\text{p}}}^{2}$ = .10, while no significant main effect of the power motive occurred, *F* < 1. As can be observed in Fig. 3, *n* Power positively predicted decisions favoring submissive-looking members, *r*(48) = .28, *p* = .045, but not submissive-looking leaders, *r*(48) = − .19, *p* = .199. While the second effect was not significant, both correlations were in the expected direction and differed significantly from each other, indicating that the manipulation was successful.

#### Other motives

Again, no significant correlation was observed between *n* Power and the explicit power motive, *r*(48) = .00, *p* = .981. In order to investigate whether the aforementioned predictive relation between *n* Power and decisions as a function of role was specific to *n* Power, we replaced this motive for the explicit power motive as continuous predictor in the ANCOVA with role (i.e., leader versus member) as repeated measure. This analysis revealed no significant effects including the explicit power motive, *F*s ≤ 1.03, *p*s ≥ .317. The implicit affiliation and achievement motives showed no such predictive relationships either, *F*s < 1, indicating that the results were specific to the power motive. The exception to this was a significant main effect of the implicit affiliation motive, *F*(1, 48) = 4.91, *p* = .031, $\eta _{{\text{p}}}^{2}$ = .09. Estimated marginal means indicated that participants relatively high in the implicit affiliation motive (i.e., *M* + 1*SD*) were more likely to select submissive faces (*M* = 58.52%, *SE* = 2.54) than participants relatively low in the affiliation motive (i.e., *M* − 1*SD; M* = 50.48%, *SE* = 2.54). As the submissive faces may have also been considered relatively friendly-looking, this may indicate that the implicit affiliation motive predicts more decisions favoring own group members being friendly looking, irrespective of their power-related function within the group. Adding the implicit affiliation motive to the main analysis with *n* Power did not change the significance of the latter motive’s predictive relation in interaction with the decision condition, *F*(1, 46) = 6.78, *p* = .012, $\eta _{{\text{p}}}^{2}$ = .13.

### Discussion

Study 2 provided a replication of Study 1. Like before, we tested whether *n* Power predicts choices based on instrumentality for getting influence over others. Specifically, the instrumentality of the different options was now manipulated based on the function of the to-be-selected individual (i.e., influential vs. not). The results again supported our hypothesis that people would select a more dominant-looking person for the instrumental function, and a more submissive-looking for the non-instrumental function.

## General discussion

Previous research has attempted to identify specific behaviors and preferences that can be predicted on the basis of implicit motives (McClelland et al. 1989; Stanton et al. 2010; Schultheiss and Brunstein 2010). Expanding on this previous research, the current studies aimed to offer a more direct test for the assumed relationship between the implicit power motive and choices based on perceived instrumentality. Specifically, with a newly developed task that allowed for the manipulation of perceived instrumentality of to-be-selected (submissive-looking/dominant-looking) leaders in a competitive intergroup context, Study 1 showed that *n* Power predicted stronger preferences for submissive-looking leaders for an out-group compared to leaders for the in-group. Study 2 replicated these findings when the selection of leaders for the in-group was contrasted with the selection of a new member of one’s in-group. These studies together point to *n* Power predicting choices specifically based on perceived instrumentality for attaining influence.

These results support the idea that behavior is generally predicted by perceived instrumentality, which is an assumption made by theoretical models regarding implicit motives (Atkinson et al. 1960; McClelland 1985; McClelland et al. 1989; Schultheiss 2001, 2008; Stanton et al. 2010) and motivation in general (Aarts and Elliot 2012). However, the present findings also suggest that the predictive relationship between implicit motives and behavior is not as strict as previously suggested. Previous research suggested that implicit motives predict preferences for specific stimuli (e.g., submissive facial expressions) and behaviors (e.g., acting dominantly and assertively). In contrast, the current studies imply that the relation between implicit motives and behavior is more flexible, and tuned to the instrumentality of the behavior in the social context. Recent studies on inter-group interactions resonate well with this idea. In these studies (Ditlmann et al. 2017), African-American participants wrote a letter to a bogus White American, in which they were asked to discuss the “history of slavery and its implications for intergroup relations today” (p. 120). As a dependent measure, researchers studied the content of these letters. Findings indicated that participants high in *n* Power used more affiliation-related imagery; indeed, it turned out that letters that contained affiliation-related imagery were rated as stronger, more impressive, and more reasonable (by White Americans). So, in line with the present findings, this study suggests that people high in *n* Power strategically wrote letters that were instrumental in making an impact on its intended recipients (Ditlmann et al. 2017)—even though the behavior itself was more affiliation-related.

Apart from the more strategic nature of implicit motives in guiding actions in an intergroup context, the present findings may have implications for theory and research on the exact nature of behaviors guides by implicit motives. As mentioned before, it has often been argued that implicit motives do not predict explicit choices (e.g., Biernat 1989; McClelland et al. 1989; Schultheiss 2001; Slabbinck et al. 2013; Spangler 1992). The current studies, however, employed a decision-making paradigm in which people made explicit choices, and findings clearly point to the possibility that implicit motives can predict such choices. We assume that implicit motives predicted these choices because participants made them based on affective responses to the faces, which are modulated by implicit motives (Schultheiss and Schiepe-Tiska 2013; Stanton et al. 2010). Thus, whereas the affective responses to the choice options entered consciousness, the implicit power motive itself might have not, and so the differential instrumentality of the options for attaining power remains implicit. Because participants are unaware of the psychological mechanisms leading up to their preference, the choice task can be considered non-declarative. Although the line of reasoning above is largely based on speculation, it is important to emphasize that in agreement with this notion, we did not find a relationship between measures of explicit motives and decisions.

Several limitations of the current studies should be mentioned. The sample sizes were relatively small (Study 1: *n* = 53; Study 2: *n* = 50), which precludes strong conclusions about the effect size of the relation between *n* Power and leader and member choices (Gelman and Carlin 2014). The current studies introduced a novel task (the leader/member choice task) and a new context manipulation (the minimal group paradigm), making it hard to derive the effect size from previous findings in implicit motive research. Notably, however, the effect in Study 1 was relatively large (*r* = .39 for the rival group choices; *r* = − .35 for the own group choices) compared to that of Study 2 (*r* = .28 for member choices; *r* = − .19 for leader choices). Replications with large samples are needed to find a more robust effect size estimate. Furthermore, future studies could examine whether the observed effect replicates in a more ecologically valid situation, as the choice task and the minimal group manipulation are somewhat contrived and the studies were run in a controlled lab setting. Finally, future studies may measure affective responses to the faces, as they likely mediate the choice results (see also Rösch et al. 2013).

To conclude, the current research attempted to extend the understanding of implicit motives’ predictive capabilities in relation to choices in an intergroup-context. We observed that the predictive strength and direction of implicit motives is conditional on the instrumentality towards the attainment of motive-related incentives. By doing so, we hope to have contributed to a generally improved understanding of how implicit motives operate.

## Electronic supplementary material

Below is the link to the electronic supplementary material.

Supplementary material 1 (DOCX 27 KB)

## Footnotes

1 A coding error resulted in uneven distribution over the different within-subjects conditions for three participants. As our dependent variable constitutes a percentage of decisions for a specific face per within-subjects condition, we chose to maintain the data of these participants regardless of this confound. Excluding these participants did not change the significance of the main hypothesized result (i.e., the interaction between group and *n* Power), *F*(1, 46) = 10.05, *p* = .003, η_p_^2^ = .18.

2 The absolute number of power motive images (*M* = 4.48, *SD* = 2.35) was again slightly right-skewed (skewness = .42, *SE* = .34) and correlated significantly with story length in words (*M* = 560.52, *SD* = 160.55), *r*(51) = .52, *p* < .001. We therefore again converted the implicit power motive score to standardized residuals after a regression for word count. For the PRF, the achievement (α = .70), power (α = .85), and affiliation (α = .73) scales all showed at least acceptable reliability. The questionnaires regarding how connected people felt with the own (α = .93) and rival (α = .85) groups again showed high reliability.
