# Supplementary material for: The implicit power motive predicts decisions in line with perceived instrumentality
Source: Motiv Emot. 2018 Mar 28;42(3):309–20. doi: 10.1007/s11031-018-9687-1 (PMC5915518; doi:10.1007/s11031-018-9687-1)
Supplement: Supplementary file 1 — Supplementary material 1 (DOCX 27 KB) [file 11031_2018_9687_MOESM1_ESM.docx]

**Supplementary Material**

The Implicit Power Motive Predicts Decisions in Line with Perceived Instrumentality

# Test-retest reliability

## Study 1

In line with the cover story of a second session occurring during which participants had to perform a competitive between-group task, participants were requested to come back to the lab on the indicated preferred date. Forty-two participants came (79.25% of the original sample; 29 females; *M*_age_ = 21.10 years, *SD*_age_ = 2.44). The average time between the two sessions was 7.93 days (*SD* = 2.40). Upon arrival, participants first completed the Picture-Story Exercise (PSE). The order of the pictures in the PSE was the same as had been randomly determined in the first session. After the PSE, participants completed an unrelated task. Finally, they filled out the Personality Research Form (PRF; achievement: α = .77; power: α = .82; affiliation: α = .75). In this questionnaire, questions were again presented in the same order as had been randomly determined during the first session.

Enabling us to match data across both sessions, participants again filled out several demographic questions (e.g., sex, age). For six participants, demographic data did not match between the two sessions, indicating either an error in determining participants’ original participant ID or participants not consistently reporting their demographics. These participants were excluded from further analyses. For the remaining participants (n = 36), we computed correlations for each all indicators from the PSE and the PRF. These correlations are reported in Table S1.

## Study 2

Participants again returned to the lab on the indicated preferred date. 48 students (96.00% of the original sample; 35 females *M*_age_ = 22.00, *SD*_age_ = 2.93) showed up. The average time between the sessions was 6.71 days (*SD* = 1.64).

The second session again consisted of a re-test of the Picture Story Exercise (PSE) and the Personality Research Form (PRF). Again, participants started with the PSE. This was followed by an unrelated task and the PRF (achievement: α = .65; power: α = .84; affiliation: α = .81). Contrary to the second session of Study 1, we now re-randomized the order of the pictures (PSE) and items (PRF). Like in Study 1, we matched data from both sessions based on demographic questions (e.g., sex, age). For one participant, demographic data did not match between sessions. Data from this participants was excluded from analyses. Analyses of the remaining data (n = 47) are presented in Table S2.

Table S1

*Means and standard deviations of and correlations between the PRF’s motive measures (PRF), unstandardized PSE motive measures, activity inhibition and word count (PSE-U), and PSE motive measures and activity inhibition standardized for word count (PSE-Z) across the first and second session of Study 1.*

| motive | session 1 | |  | session 2 | |  |  |
| --- | --- | --- | --- | --- | --- | --- | --- |
|  | *M* | *SD* |  | *M* | *SD* | *R* | *p* |
| Achievement (PRF) | 4.73 | 0.76 |  | 4.79 | 0.83 | .862 | <.001 |
| Power (PRF) | 4.53 | 0.84 |  | 4.58 | 0.80 | .885 | <.001 |
| Affiliation (PRF) | 5.08 | 0.75 |  | 5.17 | 0.70 | .827 | <.001 |
| Achievement (PSE-U) | 5.69 | 3.68 |  | 5.76 | 3.57 | .396 | .009 |
| Power (PSE-U) | 5.46 | 2.87 |  | 5.26 | 3.66 | .635 | <.001 |
| Affiliation (PSE-U) | 5.46 | 2.89 |  | 5.76 | 3.05 | .717 | <.001 |
| Activity Inhibition (PSE-U) | 4.59 | 3.92 |  | 4.32 | 3.27 | .644 | <.001 |
| Word Count (PSE-U) | 561.11 | 153.34 |  | 552.60 | 173.98 | .844 | <.001 |
| Achievement (PSE-Z) |  |  |  |  |  | .498 | .002 |
| Power (PSE-Z) |  |  |  |  |  | .631 | <.001 |
| Affiliation (PSE-Z) |  |  |  |  |  | .566 | <.001 |
| Activity Inhibition (PSE-Z) |  |  |  |  |  | .603 | <.001 |

Table S2

*Means and standard deviations of and correlations between the PRF’s motive measures (PRF), unstandardized PSE motive measures, activity inhibition and word count (PSE-U), and PSE motive measures and activity inhibition standardized for word count (PSE-Z) across the first and second session of Study 2.*

| motive | session 1 | |  | session 2 | |  |  |
| --- | --- | --- | --- | --- | --- | --- | --- |
|  | *M* | *SD* |  | *M* | *SD* | *R* | *p* |
| Achievement (PRF) | 4.65 | 0.66 |  | 4.66 | 0.59 | .858 | <.001 |
| Power (PRF) | 4.13 | 0.92 |  | 4.16 | 0.86 | .891 | <.001 |
| Affiliation (PRF) | 5.30 | 0.63 |  | 5.30 | 0.68 | .877 | <.001 |
| Achievement (PSE-U) | 5.00 | 2.24 |  | 5.29 | 2.43 | .700 | <.001 |
| Power (PSE-U) | 4.48 | 2.35 |  | 4.00 | 2.95 | .499 | <.001 |
| Affiliation (PSE-U) | 5.98 | 2.78 |  | 5.79 | 2.67 | .561 | <.001 |
| Activity Inhibition (PSE-U) | 4.54 | 3.59 |  | 4.06 | 3.52 | .585 | <.001 |
| Word Count (PSE-U) | 560.52 | 160.55 |  | 558.42 | 166.54 | .855 | <.001 |
| Achievement (PSE-Z) |  |  |  |  |  | .628 | <.001 |
| Power (PSE-Z) |  |  |  |  |  | .388 | .007 |
| Affiliation (PSE-Z) |  |  |  |  |  | .529 | <.001 |
| Activity Inhibition (PSE-Z) |  |  |  |  |  | .494 | <.001 |

*Note.* The implicit power motive included an outlier for the second session (z > 3). Excluding this participant revealed a significant correlation between the standardized implicit power motive scores of both sessions of, *r*(45) = .471, *p* = .001.

## Interpretation

In a previous discussion of the test-retest reliability of the PSE, Schultheiss and Pang (2007) concluded that the PSE is moderately stable over time, with average test-retest reliabilities of .60 when the interval between tests is one week. In general, our findings are consistent with this conclusion, with test-retest correlations of key unstandardized PSE scores (achievement, power, affiliation and activity inhibition) generally in the .40 to .70 range.

# Latencies

While our key predictions were about choice (between submissive vs. dominant-looking people), we also explored how *n* Power predicted the speed of people’s decisions. Indeed, prior work indicated that thirsty participants responded faster to drinking-related items than non-thirsty participants (Aarts, Dijksterhuis & De Vries, 2001) suggesting that people have an increased approach-response tendency to stimuli that currently represent high incentive value to them. Extending the latter idea, it may be predicted that people high in *n* Power make faster choices for submissive faces.

## Study 1

We conducted a repeated measures ANOVA across the full 2(group: own vs. rival) by 2(left: submissive vs. dominant) by 2(right: submissive vs. dominant) design with *n* Power as a continuous predictor. In preparation, all responses faster than 100 milliseconds (0.1% of trials) and longer than 4 standard deviations from the mean response time (0.7% of trials, ≥ 10,641 milliseconds) were removed to prevent outlying values or pre-meditated responses from affecting the results. This analysis revealed a significant main effect of group, *F*(1, 51) = 4.75, *p* = .034, η^2^_p_ = .09, with responses being relatively fast for the own (*M* = 2464 milliseconds, *SE* = 140) versus rival (*M* = 2594, *SE* = 146) group. Additionally, there was a significant interaction effect between left and right, *F*(1, 51) = 11.77, *p* = .001, η^2^_p_ =.19, with responses being fastest when the two presented faces were of different face types. Importantly, no significant effects were observed that involved *n* Power, *F*s ≤ 1.02, *p*s ≥ .317. Hence, no evidence was found for the idea that *n* Power predicted the speed of people’s decisions.

## Study 2

We conducted the same analysis as reported in the previous section. Again, we excluded all responses faster than 100 milliseconds (< 0.1% of trials) and longer than four standard deviations from the mean response time (1.1% of trials, ≥ 11,362 milliseconds). This analysis replicated the previously observed effect that decisions are faster for own leaders (*M* = 2489 milliseconds, *SE* = 127) versus the alternative (in this case own members; *M* = 2558, *SE* = 128), *F*(1, 48) = 4.76, *p* = .034, η^2^_p_ =.09. The effect of decisions being faster when the two faces differed from each other in type than when they were of the same type also replicated here, *F*(1, 48) = 10.3, *p* = .002, η^2^_p_ =.18. Most importantly, a significant interaction between *n* Power and role was found, *F*(1, 48) = 7.64, *p* = .008, η^2^_p_ =.14. As can be seen in Figure S1, *n* Power positively predicted decision speed to a greater extent for leaders than for members. Speculatively, this may be due to the leader condition providing information regarding instrumentality, on which basis *n* Power could predict the decisions, whereas no such information was provided in the member condition. This lack of motive-relevant information may have slowed down the decision-making process for people high in *n* Power.

*Figure S1*. Estimated marginal means of decision latency in milliseconds for decisions in the leader and member conditions as a function of *n* Power in Study 2. Error bars represent Standard Errors of the mean.

# References

Aarts, H., Dijksterhuis, A., & De Vries, P. (2001). On the psychology of drinking: Being thirsty and perceptually ready. *British Journal of Psychology*, 92, 631–642. doi:10.1348/000712601162383

Schultheiss, O. C., & Pang, J. S. (2007). Measuring implicit motives. In R. W. Robins, R. C. Fraley & R. Krueger (Eds.), *Handbook of Research Methods in Personality Psychology* (pp. 322-344). New York: Guilford.
